# 2-Chloro-12-phenyl-6,7,8,9,10,11-hexa­hydro­cyclo­octa­[*b*]quinoline

**DOI:** 10.1107/S1600536808009239

**Published:** 2008-04-10

**Authors:** Ayoob Bazgir, Ali Mohammad Astaraki

**Affiliations:** a Department of Chemistry, Islamic Azad University, Dorood Branch, Dorood 688173551, Iran

## Abstract

In the mol­ecule of the title compound, C_21_H_20_ClN, the quinoline group is nearly planar and is oriented at a dihedral angle of 77.21 (3)° with respect to the phenyl ring. The conformation of the cyclooctane ring is twist-boat. In the crystal structure, there are some weak π–π inter­actions [centroid-to-centroid distances of 3.7414 (11) and 3.8633 (12) Å] between the rings of the quinoline groups.

## Related literature

For general background, see: Kalluraya & Sreenivasa (1998 ▶); Doube *et al.* (1998 ▶); Maguire *et al.* (1994 ▶). For bond-length data, see: Allen *et al.* (1987 ▶).
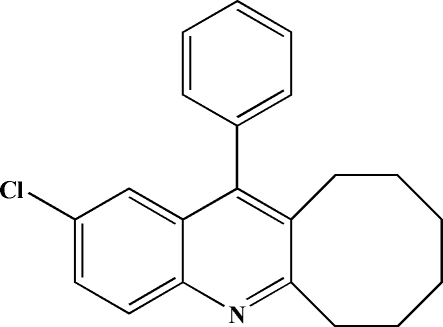


## Experimental

### 

#### Crystal data

C_21_H_20_ClN
*M* *_r_* = 321.83
Triclinic, 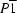

*a* = 9.837 (2) Å
*b* = 9.980 (2) Å
*c* = 10.175 (2) Å
α = 74.600 (17)°
β = 70.575 (16)°
γ = 61.829 (15)°
*V* = 823.4 (3) Å^3^
*Z* = 2
Mo *K*α radiation
μ = 0.23 mm^−1^
*T* = 298 (2) K
0.5 × 0.5 × 0.25 mm

#### Data collection

Stoe IPDSII diffractometer
Absorption correction: numerical; shape of crystal determined optically (*X-RED* and *X-SHAPE*; Stoe & Cie, 2002 ▶) *T*_min_ = 0.889, *T*_max_ = 0.949
8123 measured reflections
3801 independent reflections
3493 reflections with *I* > 2σ(*I*)
*R*_int_ = 0.045

#### Refinement

*R*[*F* ^2^ > 2σ(*F* ^2^)] = 0.046
*wR*(*F* ^2^) = 0.126
*S* = 1.03
3801 reflections
208 parameters
H-atom parameters constrained
Δρ_max_ = 0.25 e Å^−3^
Δρ_min_ = −0.19 e Å^−3^

### 

Data collection: *X-AREA* (Stoe & Cie, 2002 ▶); cell refinement: *X-AREA*; data reduction: *X-RED* (Stoe & Cie, 2002 ▶); program(s) used to solve structure: *SHELXS97* (Sheldrick, 2008 ▶); program(s) used to refine structure: *SHELXL97* (Sheldrick, 2008 ▶); molecular graphics: *ORTEP-3 for Windows* (Farrugia, 1997 ▶); software used to prepare material for publication: *WinGX* (Farrugia, 1999 ▶).

## Supplementary Material

Crystal structure: contains datablocks global, I. DOI: 10.1107/S1600536808009239/hk2449sup1.cif

Structure factors: contains datablocks I. DOI: 10.1107/S1600536808009239/hk2449Isup2.hkl

Additional supplementary materials:  crystallographic information; 3D view; checkCIF report

## Figures and Tables

**Fig. 1.**
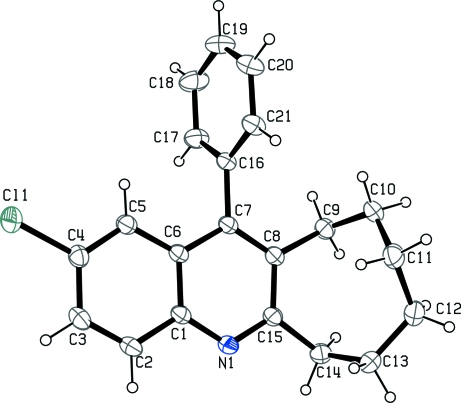
The molecular structure of the title molecule, with the atom-numbering scheme. Displacement ellipsoids are drawn at the 30% probability level.

**Fig. 2.**
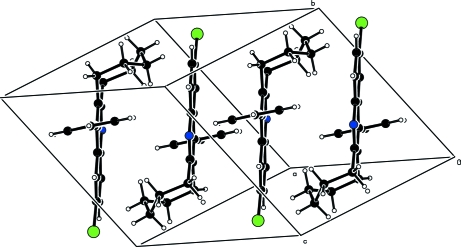
A packing diagram of (I).

## supplementary crystallographic information

### Comment

Quinoline nucleus is a backbone of many natural products and pharmacologicallly significant compounds displaying a broad range of biological activities and many functionalized quinolines are widely used as antimalarial, antiasthmatic, antiinflamatory, antibacterial, antihypertensive and tyrosine kinase PDGF-RTK inhibiting agents (Kalluraya & Sreenivasa, 1998; Doube *et al.*, 1998; Maguire *et al.*,  1994). We report herein the synthesis and crystal structure of the title compound, (I).

In the molecule of the title compound, (I), (Fig. 1) the bond lengths (Allen *et al.*,  1987) and angles are within normal ranges. Rings A (C1-C6), B (N1/C1/C6-C8/C15) and C (C16-C21) are, of course, planar, and they are oriented at dihedral angles of A/B = 0.88 (3)°, A/C = 76.76 (4)° and B/C = 77.64 (3)°. So, rings A and B are also nearly coplanar. The dihedral angle between the coplanar ring system and ring C is 77.21 (3)°.

In the crystal structure, the weak π–π interactions between the two adjacent A rings and A and B rings, with centroid-centroid distances of 3.7414 (11) Å and 3.8633 (12) Å, may be effective in the stabilization of the structure (Fig. 2).

### Experimental

A mixture of 2-amino-5-chlorophenyl(phenyl)methanone (0.23 g, 1 mmol), cyclooctanone (1.26 g, 1 mmol) and Dewax-50 W ion exchange resin (0.3 g) was heated at 353 K. After 2 h the reaction mixture was washed with ethyl acetate (10 ml). Evaporation of the solvent followed by recrystallization from ethanol to afford the pure product (yield; 0.278 g, 75%).

### Refinement

H atoms were positioned geometrically with C-H = 0.93 and 0.97 Å for aromatic and methylene H atoms, respectively, and constrained to ride on their parent atoms, with U_iso_(H) = 1.2U_eq_(C).

### Crystal data

**Table:**

C_21_H_20_Cl_1_N_1_	*Z* = 2
*M**_r_* = 321.83	*F*_000_ = 340
Triclinic, *P*1	*D*_x_ = 1.298 Mg m^−^^3^
Hall symbol: -P 1	Mo *K*α radiation λ = 0.71073 Å
*a* = 9.837 (2) Å	Cell parameters from 2086 reflections
*b* = 9.980 (2) Å	θ = 2.4–28.0º
*c* = 10.175 (2) Å	µ = 0.23 mm^−^^1^
α = 74.600 (17)º	*T* = 298 (2) K
β = 70.575 (16)º	Block, colorless
γ = 61.829 (15)º	0.5 × 0.5 × 0.25 mm
*V* = 823.4 (3) Å^3^	

### Data collection

**Table:**

Stoe IPDSII diffractometer	*R*_int_ = 0.045
rotation method scans	θ_max_ = 28.0º
Absorption correction: numericalshape of crystal determined optically [PROGRAM NAME? reference?	θ_min_ = 2.4º
*T*_min_ = 0.889, *T*_max_ = 0.949	*h* = −12→11
8123 measured reflections	*k* = −13→10
3801 independent reflections	*l* = −13→13
3493 reflections with *I* > 2σ(*I*)	

### Refinement

**Table:**

Refinement on *F*^2^	H-atom parameters constrained
Least-squares matrix: full	*w* = 1/[σ^2^(*F*_o_^2^) + (0.0607*P*)^2^ + 0.1883*P*] where *P* = (*F*_o_^2^ + 2*F*_c_^2^)/3
*R*[*F*^2^ > 2σ(*F*^2^)] = 0.046	(Δ/σ)_max_ = 0.006
*wR*(*F*^2^) = 0.126	Δρ_max_ = 0.25 e Å^−^^3^
*S* = 1.04	Δρ_min_ = −0.19 e Å^−^^3^
3801 reflections	Extinction correction: none
208 parameters	

### Special details

**Table:**

Geometry. All e.s.d.'s (except the e.s.d. in the dihedral angle between two l.s. planes) are estimated using the full covariance matrix. The cell e.s.d.'s are taken into account individually in the estimation of e.s.d.'s in distances, angles and torsion angles; correlations between e.s.d.'s in cell parameters are only used when they are defined by crystal symmetry. An approximate (isotropic) treatment of cell e.s.d.'s is used for estimating e.s.d.'s involving l.s. planes.

### Fractional atomic coordinates and isotropic or equivalent isotropic displacement parameters (Å^2^)

**Table:**

	*x*	*y*	*z*	*U*_iso_*/*U*_eq_	
Cl1	0.59962 (5)	0.96565 (4)	0.17975 (4)	0.06276 (16)	
N1	0.51106 (12)	0.54812 (13)	0.69575 (11)	0.0391 (2)	
C1	0.53821 (13)	0.64045 (14)	0.57445 (12)	0.0354 (2)	
C2	0.42557 (15)	0.79582 (15)	0.56208 (15)	0.0440 (3)	
H2	0.3377	0.8313	0.6366	0.053*	
C3	0.44417 (16)	0.89348 (15)	0.44312 (16)	0.0462 (3)	
H3	0.3693	0.9949	0.4354	0.055*	
C4	0.57823 (15)	0.83909 (14)	0.33177 (14)	0.0418 (3)	
C5	0.69071 (14)	0.69125 (14)	0.33901 (13)	0.0392 (3)	
H5	0.7787	0.659	0.2639	0.047*	
C6	0.67271 (13)	0.58733 (13)	0.46148 (12)	0.0333 (2)	
C7	0.78256 (13)	0.43037 (13)	0.47776 (12)	0.0339 (2)	
C8	0.75403 (14)	0.33702 (14)	0.60027 (12)	0.0357 (2)	
C9	0.86347 (17)	0.16885 (15)	0.62161 (14)	0.0456 (3)	
H9A	0.9111	0.1313	0.531	0.055*	
H9B	0.8003	0.1139	0.6796	0.055*	
C10	0.99695 (16)	0.13077 (18)	0.69016 (16)	0.0542 (4)	
H10A	1.0455	0.0207	0.7167	0.065*	
H10B	1.0777	0.1584	0.62	0.065*	
C11	0.94953 (18)	0.2078 (2)	0.81886 (16)	0.0549 (4)	
H11A	0.9085	0.3178	0.7909	0.066*	
H11B	1.0445	0.1765	0.8501	0.066*	
C12	0.82614 (19)	0.17430 (19)	0.94328 (16)	0.0553 (4)	
H12A	0.8568	0.1606	1.0289	0.066*	
H12B	0.8289	0.078	0.936	0.066*	
C13	0.65535 (18)	0.29655 (18)	0.95684 (14)	0.0510 (3)	
H13A	0.6551	0.3948	0.9529	0.061*	
H13B	0.5927	0.2748	1.0491	0.061*	
C14	0.57222 (16)	0.31198 (17)	0.84728 (14)	0.0470 (3)	
H14A	0.5988	0.2101	0.8307	0.056*	
H14B	0.4582	0.3605	0.8855	0.056*	
C15	0.61401 (13)	0.40276 (14)	0.70838 (12)	0.0370 (3)	
C16	0.92569 (13)	0.37293 (13)	0.35925 (12)	0.0359 (2)	
C17	0.91195 (17)	0.34403 (19)	0.23968 (14)	0.0498 (3)	
H17	0.8139	0.3575	0.2335	0.06*	
C18	1.0449 (2)	0.2949 (2)	0.12879 (17)	0.0655 (5)	
H18	1.0355	0.2746	0.0489	0.079*	
C19	1.1906 (2)	0.2758 (2)	0.13600 (18)	0.0631 (4)	
H19	1.2792	0.2421	0.0617	0.076*	
C20	1.20421 (17)	0.30678 (19)	0.25315 (18)	0.0564 (4)	
H20	1.302	0.2955	0.2578	0.068*	
C21	1.07270 (15)	0.35483 (16)	0.36480 (15)	0.0453 (3)	
H21	1.083	0.3752	0.4442	0.054*	

### Atomic displacement parameters (Å^2^)

**Table:**

	*U*^11^	*U*^22^	*U*^33^	*U*^12^	*U*^13^	*U*^23^
Cl1	0.0600 (3)	0.0434 (2)	0.0641 (3)	−0.01716 (17)	−0.01096 (18)	0.01044 (16)
N1	0.0288 (5)	0.0426 (5)	0.0396 (5)	−0.0107 (4)	−0.0059 (4)	−0.0076 (4)
C1	0.0271 (5)	0.0369 (6)	0.0400 (6)	−0.0093 (4)	−0.0084 (4)	−0.0094 (4)
C2	0.0304 (6)	0.0392 (6)	0.0524 (7)	−0.0063 (5)	−0.0053 (5)	−0.0130 (5)
C3	0.0367 (6)	0.0334 (6)	0.0607 (8)	−0.0071 (5)	−0.0131 (6)	−0.0078 (5)
C4	0.0396 (6)	0.0357 (6)	0.0486 (7)	−0.0157 (5)	−0.0132 (5)	−0.0006 (5)
C5	0.0327 (6)	0.0376 (6)	0.0426 (6)	−0.0123 (5)	−0.0068 (5)	−0.0059 (5)
C6	0.0268 (5)	0.0346 (5)	0.0377 (5)	−0.0102 (4)	−0.0084 (4)	−0.0083 (4)
C7	0.0277 (5)	0.0358 (6)	0.0366 (5)	−0.0095 (4)	−0.0080 (4)	−0.0096 (4)
C8	0.0318 (5)	0.0346 (6)	0.0391 (6)	−0.0103 (4)	−0.0104 (4)	−0.0074 (4)
C9	0.0475 (7)	0.0333 (6)	0.0455 (7)	−0.0086 (5)	−0.0103 (5)	−0.0065 (5)
C10	0.0372 (7)	0.0478 (7)	0.0542 (8)	−0.0030 (6)	−0.0116 (6)	0.0008 (6)
C11	0.0457 (7)	0.0622 (9)	0.0552 (8)	−0.0218 (7)	−0.0207 (6)	0.0034 (7)
C12	0.0548 (8)	0.0573 (9)	0.0470 (7)	−0.0206 (7)	−0.0186 (6)	0.0057 (6)
C13	0.0531 (8)	0.0526 (8)	0.0366 (6)	−0.0188 (6)	−0.0069 (5)	−0.0013 (5)
C14	0.0372 (6)	0.0509 (7)	0.0463 (7)	−0.0197 (6)	−0.0053 (5)	−0.0001 (6)
C15	0.0301 (5)	0.0413 (6)	0.0387 (6)	−0.0142 (5)	−0.0091 (4)	−0.0050 (5)
C16	0.0308 (5)	0.0325 (5)	0.0378 (6)	−0.0083 (4)	−0.0052 (4)	−0.0085 (4)
C17	0.0420 (7)	0.0655 (9)	0.0450 (7)	−0.0231 (6)	−0.0054 (5)	−0.0178 (6)
C18	0.0635 (10)	0.0914 (13)	0.0468 (8)	−0.0362 (9)	0.0029 (7)	−0.0309 (8)
C19	0.0472 (8)	0.0739 (11)	0.0574 (9)	−0.0243 (8)	0.0123 (7)	−0.0265 (8)
C20	0.0323 (6)	0.0617 (9)	0.0695 (9)	−0.0167 (6)	−0.0022 (6)	−0.0190 (7)
C21	0.0348 (6)	0.0471 (7)	0.0513 (7)	−0.0118 (5)	−0.0087 (5)	−0.0152 (6)

### Geometric parameters (Å, °)

**Table:**

C1—N1	1.3612 (16)	C11—H11A	0.97
C1—C6	1.4153 (16)	C11—H11B	0.97
C1—C2	1.4194 (17)	C12—C13	1.526 (2)
C2—C3	1.358 (2)	C12—H12A	0.97
C2—H2	0.93	C12—H12B	0.97
C3—C4	1.4051 (19)	C13—C14	1.527 (2)
C3—H3	0.93	C13—H13A	0.97
C4—C5	1.3644 (18)	C13—H13B	0.97
C4—Cl1	1.7405 (14)	C14—C15	1.5075 (18)
C5—C6	1.4143 (17)	C14—H14A	0.97
C5—H5	0.93	C14—H14B	0.97
C6—C7	1.4255 (16)	C15—N1	1.3217 (17)
C7—C8	1.3777 (17)	C16—C17	1.3836 (18)
C7—C16	1.4969 (16)	C16—C21	1.3890 (18)
C8—C15	1.4353 (17)	C17—C18	1.390 (2)
C8—C9	1.5096 (17)	C17—H17	0.93
C9—C10	1.534 (2)	C18—C19	1.378 (3)
C9—H9A	0.97	C18—H18	0.93
C9—H9B	0.97	C19—C20	1.370 (2)
C10—C11	1.521 (2)	C19—H19	0.93
C10—H10A	0.97	C20—C21	1.3857 (19)
C10—H10B	0.97	C20—H20	0.93
C11—C12	1.524 (2)	C21—H21	0.93
			
N1—C1—C6	122.66 (11)	H11A—C11—H11B	107.4
N1—C1—C2	117.98 (11)	C11—C12—C13	115.65 (13)
C6—C1—C2	119.36 (11)	C11—C12—H12A	108.4
C3—C2—C1	121.00 (12)	C13—C12—H12A	108.4
C3—C2—H2	119.5	C11—C12—H12B	108.4
C1—C2—H2	119.5	C13—C12—H12B	108.4
C2—C3—C4	119.00 (12)	H12A—C12—H12B	107.4
C2—C3—H3	120.5	C12—C13—C14	116.62 (13)
C4—C3—H3	120.5	C12—C13—H13A	108.1
C5—C4—C3	122.25 (12)	C14—C13—H13A	108.1
C5—C4—Cl1	119.37 (10)	C12—C13—H13B	108.1
C3—C4—Cl1	118.37 (10)	C14—C13—H13B	108.1
C4—C5—C6	119.61 (11)	H13A—C13—H13B	107.3
C4—C5—H5	120.2	C15—C14—C13	114.79 (11)
C6—C5—H5	120.2	C15—C14—H14A	108.6
C5—C6—C1	118.77 (11)	C13—C14—H14A	108.6
C5—C6—C7	123.61 (10)	C15—C14—H14B	108.6
C1—C6—C7	117.63 (11)	C13—C14—H14B	108.6
C8—C7—C6	119.53 (10)	H14A—C14—H14B	107.5
C8—C7—C16	122.32 (10)	N1—C15—C8	123.17 (11)
C6—C7—C16	118.15 (10)	N1—C15—C14	114.02 (11)
C7—C8—C15	118.28 (11)	C8—C15—C14	122.80 (11)
C7—C8—C9	121.86 (11)	C17—C16—C21	118.98 (11)
C15—C8—C9	119.85 (11)	C17—C16—C7	120.43 (11)
C8—C9—C10	115.07 (12)	C21—C16—C7	120.52 (11)
C8—C9—H9A	108.5	C16—C17—C18	119.87 (13)
C10—C9—H9A	108.5	C16—C17—H17	120.1
C8—C9—H9B	108.5	C18—C17—H17	120.1
C10—C9—H9B	108.5	C19—C18—C17	120.65 (15)
H9A—C9—H9B	107.5	C19—C18—H18	119.7
C11—C10—C9	116.56 (12)	C17—C18—H18	119.7
C11—C10—H10A	108.2	C20—C19—C18	119.70 (13)
C9—C10—H10A	108.2	C20—C19—H19	120.2
C11—C10—H10B	108.2	C18—C19—H19	120.1
C9—C10—H10B	108.2	C19—C20—C21	120.16 (14)
H10A—C10—H10B	107.3	C19—C20—H20	119.9
C10—C11—C12	116.04 (14)	C21—C20—H20	119.9
C10—C11—H11A	108.3	C20—C21—C16	120.63 (13)
C12—C11—H11A	108.3	C20—C21—H21	119.7
C10—C11—H11B	108.3	C16—C21—H21	119.7
C12—C11—H11B	108.3	C15—N1—C1	118.73 (10)
			
N1—C1—C2—C3	−178.99 (12)	C10—C11—C12—C13	−98.63 (17)
C6—C1—C2—C3	0.70 (19)	C11—C12—C13—C14	70.55 (19)
C1—C2—C3—C4	−0.6 (2)	C12—C13—C14—C15	−80.42 (17)
C2—C3—C4—C5	0.0 (2)	C7—C8—C15—N1	0.29 (18)
C2—C3—C4—Cl1	179.69 (11)	C9—C8—C15—N1	−178.45 (11)
C3—C4—C5—C6	0.7 (2)	C7—C8—C15—C14	−179.14 (11)
Cl1—C4—C5—C6	−179.07 (9)	C9—C8—C15—C14	2.13 (18)
C4—C5—C6—C1	−0.59 (18)	C13—C14—C15—N1	−96.60 (14)
C4—C5—C6—C7	178.98 (11)	C13—C14—C15—C8	82.88 (16)
N1—C1—C6—C5	179.60 (10)	C8—C7—C16—C17	103.88 (15)
C2—C1—C6—C5	−0.07 (17)	C6—C7—C16—C17	−75.90 (16)
N1—C1—C6—C7	0.00 (17)	C8—C7—C16—C21	−79.21 (16)
C2—C1—C6—C7	−179.67 (11)	C6—C7—C16—C21	101.02 (14)
C5—C6—C7—C8	−179.10 (11)	C21—C16—C17—C18	1.2 (2)
C1—C6—C7—C8	0.48 (16)	C7—C16—C17—C18	178.19 (15)
C5—C6—C7—C16	0.69 (17)	C16—C17—C18—C19	−0.6 (3)
C1—C6—C7—C16	−179.74 (10)	C17—C18—C19—C20	−0.5 (3)
C6—C7—C8—C15	−0.62 (17)	C18—C19—C20—C21	1.0 (3)
C16—C7—C8—C15	179.61 (10)	C19—C20—C21—C16	−0.3 (2)
C6—C7—C8—C9	178.09 (11)	C17—C16—C21—C20	−0.8 (2)
C16—C7—C8—C9	−1.68 (18)	C7—C16—C21—C20	−177.74 (13)
C7—C8—C9—C10	91.27 (15)	C8—C15—N1—C1	0.19 (18)
C15—C8—C9—C10	−90.04 (15)	C14—C15—N1—C1	179.66 (10)
C8—C9—C10—C11	46.24 (18)	C6—C1—N1—C15	−0.33 (18)
C9—C10—C11—C12	59.62 (18)	C2—C1—N1—C15	179.34 (11)
